# Unconventional High-Performance Laser Protection System Based on Dichroic Dye-Doped Cholesteric Liquid Crystals

**DOI:** 10.1038/srep42955

**Published:** 2017-02-23

**Authors:** Wanshu Zhang, Lanying Zhang, Xiao Liang, Le Zhou, Jiumei Xiao, Li Yu, Fasheng Li, Hui Cao, Kexuan Li, Zhou Yang, Huai Yang

**Affiliations:** 1 Department of Materials Physics and Chemistry, School of Materials Science and Engineering, University of Science and Technology Beijing, Beijing 100083, P. R. China; 2 Department of Materials Science and Engineering, College of Engineering, Peking University, Beijing 100871, P. R. China; 3 Key Laboratory of Polymer Chemistry and Physics of Ministry of Education, Peking University, Beijing 100871, P. R. China; 4 Department of Chemistry, Dalian Medical University, Dalian 116044, P. R. China; 5 Department of Applied Statistics and Science, Xijing University, Xian 710123, P. R. China

## Abstract

High-performance and cost-effective laser protection system is of crucial importance for the rapid advance of lasers in military and civilian fields leading to severe damages of human eyes and sensitive optical devices. However, it is crucially hindered by the angle-dependent protective effect and the complex preparation process. Here we demonstrate that angle-independence, good processibility, wavelength tunability, high optical density and good visibility can be effectuated simultaneously, by embedding dichroic anthraquinone dyes in a cholesteric liquid crystal matrix. More significantly, unconventional two-dimensional parabolic protection behavior is reported for the first time that in stark contrast to the existing protection systems, the overall parabolic protection behavior enables protective effect to increase with incident angles, hence providing omnibearing high-performance protection. The protective effect is controllable by dye concentration, LC cell thickness and CLC reflection efficiency, and the system can be made flexible enabling applications in flexible and even wearable protection devices. This research creates a promising avenue for the high-performance and cost-effective laser protection, and may foster the development of optical applications such as solar concentrators, car explosion-proof membrane, smart windows and polarizers.

Laser protection materials have sparked enormous interest from both the military and commercial perspectives for their remarkable abilities in the protection of human eyes, optical sensors, satellites and aircraft pilots^1^,^2^,^3^. To date, significant effort has been focused on the absorption-reflection complex system that combines the virtues of reflection system (good visibility) and absorption system (high optical density)^4^,^5^. However, there are currently two main problems that severely hamper its practical applications, which are the angle-dependent protective behaviors and the inevitable complex process of multilayer deposition under high temperature and vacuum^6^. In light of these deficiencies, it is highly desirable to develop an advanced complex system that can intergrate angle-independence, good processibility, tunable wavelength, high optical density (OD) and good visibility into one single film simultaneously^7^. Here we propose a complex system of dichroic anthraquinone dye-doped cholesteric liquid crystals that can fulfill all the aforementioned requirements in a single film. More significantly, unconventional two-dimensional parabolic protective effect is obtained, i.e., OD increases with incident angles thus offering omnibearing high-performance protection.

Liquid crystal (LC)-based protection materials have been extensively studied and reported by many groups for over 20 years in both linear and nonlinear laser protections. For example, Wu, C. S. *et al*.^8^ reported high-performance LC-related switchable polarizers for the protection of human eyes and optical sensors, which exhibited a clear state under the no-threat condition and quickly switched to a linear polarizer state with high extinction under the threat condition. Tutt, L. W. *et al*.^9^ reported that LCs possessed extraordinary large optical nonlinearities and occupied an important niche in nonlinear optics due to their unique physical and optical properties such as broadband birefringence, self-assembly ability and good compatibility with other optoelectronic technology platforms. As the most representive LCs, cholesteric liquid crystals (CLCs) represent fascinating prospect in laser protection owing to their inherent self-organized periodic helical superstructures within a “green”, efficient and cost-effective approach^10^. Being in accordance with the Bragg regime, reflection wavelength centered at λ_p_ = nPcosθ with a bandwidth of Δλ_p_ = Δn·p, where n is the average refractive index, P is the helical pitch, θ is the incident angle (between the incident light and the normal direction, Fig. 1a)^11^,^12^ and Δn is birefringence. The reflection wavelength of CLCs can be precisely controlled by modulating the helical pitch closely related to the chiral dopant concentrations^13^. However, the ubiquitous angle-dependent behavior (defined as OD decreasing with incident angles) of reflection protection system is also the bottleneck problem of CLCs that hinders their promising application in laser protection. As shown in Fig. 1b, as θ increases, OD first slightly increases then reduces dramatically, hence, the protective effect is jeopardized. This behavior is the joint effect of transmittance changes resulted from Bragg reflection and oblique incidence that with θ increasing, λ_p_ blueshifts resulting in the related transmittance (t_λ_) first remaining constant (in the bandwidth region) then increasing distinctly (out of bandwidth region) and the oblique incidence leading to the related transmittance (t_o_) slightly declining. As a consequence, the integral transmittance firstly slightly decreases then dramatically increases, i.e., OD first increases then decreases that the protective effect of CLCs is angle-dependent.

To this end, herein we utilize dichroic dyes as a functional component to be doped in CLCs. As we know, positive dichroic dyes which align the same as the nematic liquid crystal host by cooperative motion exhibit anisotropic absorption behaviors with the primary absorption dipole along the molecular long axis and the secondary absorption dipole along the molecular short axis^14^,^15^,^16^,^17^. As θ increases, the source light initially along the secondary absorption dipole is gradually prone to be parallel with the the primary absorption dipole. Hence, as shown in Fig. 1c, the dichroic dyes exhibit gradually ascending ODs with an overally unconventional parabolic protection behavior and can compensate for the CLC reflection decrease (Fig. 1c)^18^. The combination of CLCs and dichroic dyes with the absorption wavelength in match with that of CLC reflection leads to optimal OD values while maintains self-organization behaviors and good visibility of CLCs. In addition, the angle-dependent behavior of CLCs is obviously eliminated by doping dichroic dyes due to their compensation function of ascending ODs and the unprecedented two-dimensional parabolic protection with enhanced ODs is realized by synergistic effect (Fig. 1d)^19^.

## Results

### Properties of the doped anthraquinone dyes

**Figure 1:**
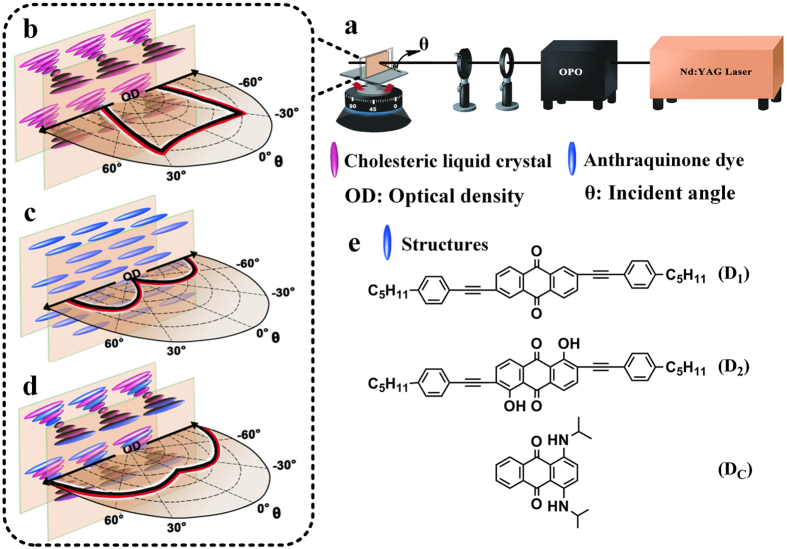
Experimental setup, schematic digram and chemical structures of AQ dyes used in this study. (**a**) Schematic experimental setup of the laser protection measurement; (**b**–**d**) Enlarged illustrations of the molecular arrangements with the polar plots of OD versus θ for samples of (**b**) CLCs, (**c**) Dichroic dyes, (**d**) Dichroic dye-doped CLCs; (**e**) The chemical structures of the doped dichroic dyes D_1_, D_2_ and the contrastive dye D_C_.

Anthraquinones (AQs) are selected as the parent attributed to their excellent photostability and wide color selectivity^20^. In order to comprehensively illustrate the function of dichroic dyes that eliminates the angle-dependent behaviors of CLCs, three dyes in which D_1_, D_2_ are dichroic^21^, and D_C_ is designed discoid for comparison^22^ based on the AQ parent are synthesized (Fig. 1e, Supplementary Figs S1–S6). From the polarized absorption spectra, it can be observed that dichroic ratios (D_A_) and order parameters (S_A_) of dyes D_1_, D_2_ and D_C_ are 13.26, 12.17, 1.57 and 0.81, 0.79, 0.16, respectively (Supplementary Fig. S7). High values of D_A_ and S_A_ possessed by D_1_ and D_2_ indicate they exhibit superior dichroic properties caused by the high molecular aspect ratios of AQ dyes^23^. On the contrary, dye D_C_ shows low value of D_A_ and S_A_ limited by its disc-like structure^15^. Besides influence the dichroic property of dyes, the introduction of moieties at the α-positions along with triple bonds and long alkyl chains endows dichroic dyes D_1_ and D_2_ with outstanding solubility in nematic LC E7^21^. Meantime, dichroic dyes D1 and D2 both have good UV stabilities benchmarking on the sample photopolymerization condition (5.0 mW/cm^2^, 600.0 s) that upon 50.0 mW/cm^2^ UV irradiation, D1 and D2 can maintain for 1800.0 s and 7200.0 s, respectively (Supplementary Fig. S8). Compared with D1, D2 exhibits 3 times better UV stability due to the absorption factor that the introduced –OH radicals red-shifts the longest absorption tail from the ultraviolet (D1, 370 nm) to the visible regions (D2, 487 nm) with significantly weaker absorption around the UV irradiation wavelength (365 nm)^24^,^25^. Moreover, introduction of different electron-donating radicals (-NH->-OH>-H) enables trichromatic (blue, red, yellow) dyes that collectively shield the light over a wide range of 250–700 nm (Supplementary Fig. S9)^26^.

### Parabolic Laser protection characterization

**Figure 2:**
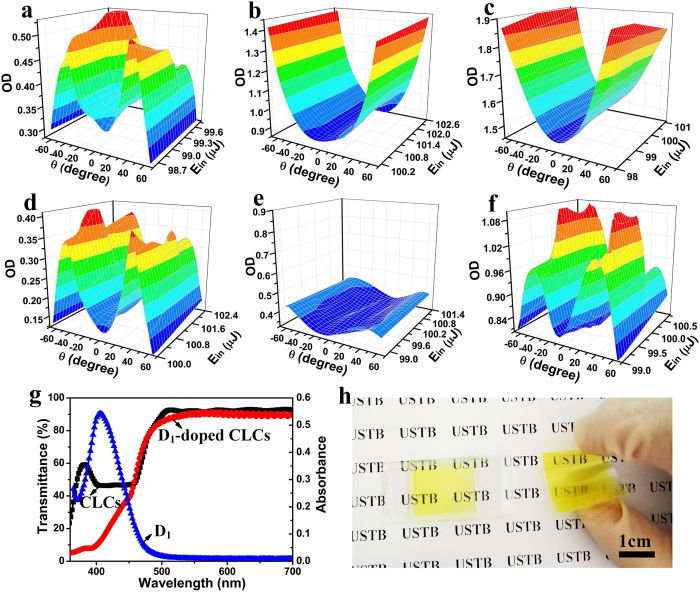
Laser protection measurements, UV-vis spectra and photographs for D_1_ series. (**a**–**f**) Three-dimensional figures of OD measurements as a function of θ and E_in_ (Power density of 3.18 × 10^−3^ J/cm^2^) of (**a**–**c**) D_1_ series and (**d**–**f**) D_C_ series. (**a**,**d**) CLC reflection samples. (**b**,**e**) Dye absorption samples. (**c**,**f**) Dye-doped CLC samples. (**g**) The UV-Vis spectra of the three samples of D_1_ series. (**h**) The digital photograph of the D_1_-doped CLC sample in LC cell (left) and flexible films (right). The LC cells are 20 μm thick. All the figures are the results removing the influence of empty LC cells (Supplementary Fig. S11).

The OD values of CLCs, wavelength matched dyes and the corresponding complex systems (Supplementary Fig. S10 and Supplementary Tables S1–S3) as a function of incident angle (θ) and energy (E_in_) are combined to illustrate the synergistic effect between CLCs and dichroic dyes in improving the OD values of the complex system. As presented in Fig. 2a, the OD value of CLCs with reflection wavelength centered at 420 nm shows typical angle-dependent behavior that OD value firstly raises then dramatically declines in measured angle region. For instance, as the angle is between 0 and 40°, the OD value increases from 0.37 to 0.54. As the angle is between 40 and 65°, the OD value decreases straightly to 0.27. In contrast to CLCs, the OD value of the wavelength matched dichroic dye D_1_ shows parabolic protection with an ascending OD trend of 0.90 to 1.42 with θ from 0° to 65° (Fig. 2b). By the combination of CLCs and dichroic dyes, it can be obviously observed that the OD value is remarkably raised in the measured angle region due to the synergistic effect that D_1_ aligning as the CLC host displays gradually ascending ODs with increasing θ and compensates for the OD value decrease of CLCs (Fig. 2c). The angle-dependent behavior of CLCs is eliminated while unprecedented parabolic protection behavior appears due to the dichroism of the doped dyes. To further demonstrate the unique compensation capability of dichroic dyes, the OD value of CLCs with reflection wavelength centered at 600 nm, wavelength matched dyes D_C_ and the corresponding complex systems as a function of incident angle and energy is also investigated. As seen in Fig. 2d,e and f, although the OD value of the complex system is dramatically enhanced in the measured region of the angle, while the angle-dependent behavior of CLCs is retained due to the inferior dichroism of D_C_ that it shows practically invariable OD values with increasing θ, incapable of compensating for the OD value decrease of CLCs. Moreover, another dichroic dye D_2_ is utilized to prove the universality of the complex system facilitated by the precisely tunable wavelength of CLCs to be in match with the doped dyes. The D_2_-doped CLC complex system also eliminates the angle-dependent behavior of CLCs and displays the unconventional parabolic protection with enhanced OD values by synergistic effect as the dichroic D_1_ series (Supplementary Fig. S12). Meantime, the wavelength matched complex systems with enhanced ODs simultaneously maintain the good visibility of CLCs (Fig. 2g and Supplementary Fig. S13). For example, the visibility of D_1_-doped CLC sample is as high as 90.3% with the corresponding digital photograph in LC cell shown in Fig. 2h (left), which can be further made flexible with promising advantages of flexibility, shape diversity and light weight. These properties enable the dye-doped CLC complex system further with applications in flexible and even wearable laser protection devices (Fig. 2h, right). Finally, the protective effect of the proposed dye-doped CLC complex system is in relatively good stability that with the incident energy (E_in_) increasing from 100 to 1000 μJ, OD values can remain stable for both D1 and D2 series (Supplementary Fig. S14).

### Factors for protection effect enhancement

**Figure 3:**
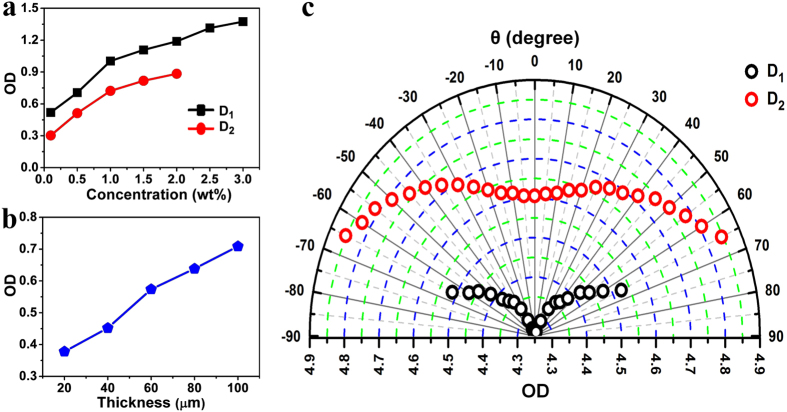
Influence factors on OD values. (**a**) ODs of D_1_ and D_2_ absorption with different concentrations in nematic LCs HNG726200-100 measured at normal angle. (**b**) ODs of CLC reflection in LC cells of different thicknesses measured at normal angle. (**c**) Polar plot of OD as a function of θ for the dye-doped double-handed CLC samples of D_1_ and D_2_ by washout/refill method.

To further improve the OD value of the proposed complex system, the effect of doped dye concentration, thickness of LC cell and CLC reflection efficiency on the OD value is explored. As shown in Fig. 3a, the OD values of dye absorption increase with the concentrations and D_1_ exhibits a higher OD value than D_2_ under the same condition of concentration and cell thickness in accordance with the absorbance spectra (Supplementary Fig. S15). In princple, the OD value linearly increases with the augment of the LC cell thickness (Fig. 3b). However, the good visibility of great importance to the devices, especially for the protection of human eyes, cannot be retained as the thickness of LC cell is over 60 μm beacuse excellent orientation of CLCs can be hardly obtained in this condition (Supplementary Figs S16 and S17). As we know, the reflection efficiency of double-handed CLCs is higher than that of single-handed CLCs due to the chiraloptical selectivity^27^,^28^. The washout/refill approach is selected to fabricate monolayer CLC cell reflecting double-handed light simultaneously to enhance the OD value (for details see the Supplementary Tables S5 and S6 and Supplementary Figs S20 and S21)^29^. The dye-doped double-handed CLC complex systems for D_1_ and D_2_ both exhibit parabolic protection behaviors with OD values of 4.60–4.85 and 4.25–4.53 from 0° to 65° in luminous transmittance of 26.9% and 40.9%, respectively (Fig. 3c and Supplementary Figs S22 and S23). Compared to the single-handed complex systems (Supplementary Table S4, Supplementary Figs S18 and S19), OD values of the double-handed systems are remarkably enhanced that taking θ = 0° for instance, the OD value growth is as high as 34.5% and 95.0% for D_1_ and D_2_ series, respectively. Furthermore, the proposed dye-doped CLC complex system also has promising prospect in notch filters in that the transmittance ratio of the pass- and stop-bands is as high as 10709.08 (D1 series for example) and it simultaneously outclasses the traditional notch filters in the good processibility with no need of complex structural design and multilayer stacking^30^.

## Discussion

This work explores an unprecedented two-dimensional parabolic laser protection system of dichroic dye-doped CLCs with advantages of angle-independence, good processibility, tunable wavelength, high optical density and good visibility simultaneously intergrated. Additionally, this protection system is applicable to both the continuous wave and pulsed lasers and cost-effective with great envisions to create millions of economic and social benefits. Owing to the intrinsic feature of CLCs, there is great potentiality for the protective effect improvement such as multiple stacking^31^, wideband protection^32^ and stimuli-controllable protection^33^,^34^,^35^. This research creates a promising avenue for the high-performance and cost-effective laser protection with great potentials in flexible and even wearable protection devices, broadens the application of CLCs with their inherent angle-dependent behaviors eliminated, and thus may pave a new way for optical applications such as solar concentrators, car explosion-proof membrane, smart windows and polarizers.

## Methods

### Characterization

^1^H NMR (400 MHz) and ^13^C NMR (100 MHz) spectra were measured in CDCl_3_ on Bruker-ARX400 spectrometer at room temperature using tetramethylsilane (TMS) as internal standard. All MALDI-TOF-MS spectra were measured on a Shimadzu AXIMA-CFR mass spectrometer. The operation was performed at an accelerating potential of 20 KV by a linear positive ion mode with dithranol as a matrix. UV-vis spectra were recorded on a Perkin /Elmer Lambda 950 UV-VIS-NIR spectrophotometer. Polarized optical microscope (POM) observations were performed with Zeiss Axio Scope A1 Microscope. Elemental analysis was carried out with a Perkin-Elmer Analyzer 2400 with an accuracy of ±0.3%. Morphologies of the polymer networks were observed by using scanning electron microscopy (SEM, Zeiss EVO18, Germany).

### Sample manufacturing

Homogeously aligned LC cells were prepared by putting together two ITO glass plates coated with alignment layer of polyimide. The alignment layer was spin-coated, thermally cured and subsequently unidirectionally buffered with a velvet cloth to ensure alignment of the LC director at the surfaces. The plates were cemented together and separated with spacers of 20 to 100 μm to assure a well-defined LC layer thickness. Subsequently, the empty cells were filled with the LC mixtures by capillary action at 60 degree. The samples were cooled to room temperature and polymerized under 365 nm UV light of 5.0 mW/cm^2^ intensity for 10.0 minutes.

### Dichroism characterization

The dichroism measurements of anthraquinone dyes were performed using Perkin/Elmer Lambda 950 UV-VIS-NIR spectrophotometer equipped with Glan air-space calcite polarizers in both sample and reference beam. The dyes were doped in nematic LC E7 and measured in homogeously aligned LC cells of 20 μm thickness. Dichroic ratios (D_A_) and order parameters (S_A_) were caculated from the equations below:


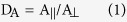



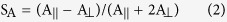



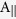
 and 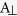 were the absorption spectra at parallel and perpendicular to the liquid crystal alignment, respectively.

### Washout/Refill Method

The dye-doped double-handed CLC complex samples were achieved by washout/refill method with the following procedure. At first, the cells containing samples with a left-handed helical structure (LHHS) were irradiated with UV light (365 nm, 5.0 mW/cm^2^) for 10.0 min for polymerization purposes. Following that, the cells were immersed in cyclohexane for about 5 days to remove the nonreactive LCs. Then the cells were kept in a vacuum chamber at 50.0 °C for about 3.0 h. Thus, the polymer network with a LHHS was obtained. Finally, the cells containing the polymer network were refilled with right-handed CLC and dye mixtures by vacuum filling process, hence, the dye-doped double-handed CLC complex samples were obtained.

### Laser protection measurement

An optical parametric oscillator (PremiScan/240/MB-ULD OPO, Spectra-Physics, 10 Hz repetition and 8–12 ns duration) pumped by the third-harmonic light of a Nd: YAG laser (Lab170, Spectra-Physics) was used as a light source for the laser protection experiments. To be in consistence with the actual application conditions for laser protection materials, the light source is not linearly polarized and no additional polarizer is used. The beam intensity was controlled using neutral density filters and measured with a pyroelectrical energy meter (Coherent EMP 2000). The pump beam was focused into a spot of 2 mm diameter and the laser wavelengths used are 420 nm, 500 nm and 600 nm. The working range of the incident laser energy is below 1000 μJ. Samples were set perpendicularly to the laser incident direction with the initial incident angle of 0° and rotate clockwise (0° to 65°) or anticlockwise (0° to −65°) horizontally. Every 5 degree of rotation, data of incident and transmitted laser energy were collected, and optical density (OD) was calculated with the following equation:


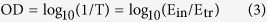



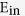
 and 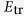 were the incident and transmitted laser energy, respectively.

## Additional Information

**How to cite this article:** Zhang, W. *et al*. Unconventional High-Performance Laser Protection System Based on Dichroic Dye-Doped Cholesteric Liquid Crystals. *Sci. Rep.* **7**, 42955; doi: 10.1038/srep42955 (2017).

**Publisher's note:** Springer Nature remains neutral with regard to jurisdictional claims in published maps and institutional affiliations.

## Supplementary Material

Supplementary Information

## References

[b1] Spangler C. W. Recent development in the design of organic materials for optical power limiting. J. Mater. Chem. 9, 2013–2020 (1999).

[b2] Huang Z. . Low-cost, large-scale, and facile production of Si nanowires exhibiting enhanced third-order optical nonlinearity. ACS Appl. Mater. Interfaces 4, 1553–1559 (2012). 22329903 10.1021/am201758z

[b3] Zhou G., Wong W. Y., Poon S. Y., Ye C. & Lin Z. Symmetric versus unsymmetric platinum (II) bis (aryleneethynylene)s with distinct electronic structures for optical power limiting/optical transparency trade-off optimization. Adv. Funct. Mater. 19, 531–544 (2009).

[b4] Muric B. D., Pantelic D. V., Vasijevic D. M., Savic-Sevic S. N. & Jehenkovic B. M. Application of tot’hema eosin sensitized gelatin as a potential eye protection filter against direct laser radiation. Curr. Appl. Phys. 16, 57–62 (2016).

[b5] Muranova G. A., Videnichev D. A. & Mikhaĭlov A. V. Multispectral optical coatings for protection from laser radiation. J. Opt. Technol. 79, 236–240 (2012).

[b6] Taracheva E., Yulin S., Feigl T. & Kaiser N. High-Performance Multilayer Coatings for 106 nm. Proc. SPIE. 6705, 67050Y (2007).

[b7] Ritt G., Walter D. & Eberle B. Reaearch on laser protection- an overview of 20 years of activities at fraunhofer IOSB. Proc. SPIE. 8896, 88960G (2013).

[b8] Wu C. S. & Wu S. T. Liquid-crystal-based switchable polarizers for sensor protection. Appl. Opt. 34, 7221–7227 (1995). 21060588 10.1364/AO.34.007221

[b9] Tutt L. W. & Boggess T. F. A review of optical limiting mechanisms and devices using organics, fullerenes, semiconductors and other materials. Prog. Quant. Electron. 17, 299–338 (1993).

[b10] Ha N. Y. . Fabrication of a simultaneous red-green-blue reflector using single-pitched cholesteric liquid crystals. Nature 7, 43–47 (2008). 10.1038/nmat2045 17994028

[b11] Motiv M. Cholesteric liquid crystals with a broad light reflection band. Adv. Mater. 24, 6260–6276 (2012). 23090724 10.1002/adma.201202913

[b12] Geng Y. . High-fidelity spherical cholesteric liquid crystal Bragg reflectors generating unclonable patterns for secure authentication. Sci. Rep. 6, 26840 (2016). 27230944 10.1038/srep26840 PMC4882534

[b13] Zhang L. . Polymeric infrared reflective thin films with ultra-broad bandwidth, Liq. Cryst, 43, 750–757 (2016).

[b14] Bruijnaers B. J., Schenning A. P. H. J. & Debije M. G. Capture and concentration of light to a spot in plastic lightguides by circular luminophore arrangements. Adv. Opt. Mater. 3, 257–262 (2015).

[b15] Debije M. G., Menelaou C., Herz L. M. & Schenning A. P. H. J. Combining positive and negative dichroic fluorophores for advanced light management in luminescent solar concentrators. Adv. Optical Mater. 2, 687–693 (2014).

[b16] Yu H. Recent advances in photoresponsive liquid-crystalline polymers containing azobenzene chormophores. J. Mater. Chem. C. 2, 3047–3054 (2014).

[b17] Yu H., Kobayashi T. & Yang H. Liquid-crystalline ordering helps block copolymer self-assembly. Adv. Mater. 23, 3337–3344 (2011). 21910267 10.1002/adma.201101106

[b18] Menelaou C. . Rapid energy transfer enabling control of emission polarization in perylene bisimide donor-acceptor triads. J. Phys. Chem. Lett. 6, 1170–1176 (2015). 26262968 10.1021/acs.jpclett.5b00183

[b19] Sun H., Xu Z. & Gao C. Multifunctional, ultra-flyweight, synergistically assembled carbon aerogels. Adv. Mater. 25, 2554–2560 (2013). 23418099 10.1002/adma.201204576

[b20] Iwanaga H. Development of highly soluble anthraquinone dichroic dyes and their application to three-layer guest-host liquid crystal displays. Materials 2, 1636–1661 (2009).

[b21] Zhang W. . Synthesis and characterisation of liquid crystalline anthraquinone dyes with excellent dichroism and solubility. Liq. Cryst. 43, 1307–1314 (2016).

[b22] Ramrao K. U., Ramkumar C. A., Anant N. A. & Ramanuja A. N. Phase-transfer catalyzd N-monoalkylation of aminoanthraquinones. Syn. Commun. 21, 1129–1135 (1991).

[b23] Park S., Park J., Lee S. & Park J. New dyes based on anthraquinone derivatives for color filter colorants. J. Nanosci. Nanotechnol. 14, 6435–6437 (2014). 25936132 10.1166/jnn.2014.8809

[b24] Chen R., An Z., Wang W., Chen X. & Chen P. Improving UV stability of tolane-liquid crystals in photonic applications by the ortho fluorine substitution. Opt. Mater. Express 6, 97–105 (2016).

[b25] Lin P. T., Wu S. T., Chang C. Y. & Hsu C. S. UV stability of high birefirngence liquid crystals. Mol. Cryst. Liq. Cryst. 411, 1285–1295 (2004).

[b26] Son H. J. . Synthesis of fluorinated polythienothiophene-co-benzodithiophenes and effect of fluorination on the photovoltaic properties. J. Am. Chem. Soc. 133, 1885–1894 (2011). 21265569 10.1021/ja108601g

[b27] Xiang J. . Electrically tunable selective reflection of light from ultraviolet to visible and infrared by heliconical cholesterics. Adv. Mater. 27, 3014–3018 (2015). 25821155 10.1002/adma.201500340 PMC4683668

[b28] Varanytsia A. . Tuable lasing in cholesteric liquid crystal elastomers with accurate measurements of strain. Sci. Rep. 5, 17739 (2015). 26634336 10.1038/srep17739 PMC4669456

[b29] Guo J. . Polymer stabilized liquid crystal films reflecting both right- and left-circularly polarized light. Appl. Phys. Lett. 93, 201901 (2008).

[b30] Lim S., Shih S. & Wager J. F. Design and fabrication of a double bandstop rugate filter grown by plasma-enhanced chemical vapor depostion. Thin Solid Films 277, 144–146 (1996).

[b31] Ha N. Y., Jeong S. M., Nishimura S. & Takezoe H. Color- and reflectance-tuable multiple reflectors assembled from three polymer films. Adv. Mater. 22, 1617–1621 (2010). 20496391 10.1002/adma.200903415

[b32] Broer D. J., Lub J. & Mol G. N. Wide-band reflective polarizers from cholesteric polymer networks with a pitch gradient. Nature 378, 467–469 (1995).

[b33] Hu W. . Electrically controllable selective reflection of chiral nematic liquid crystal/chiral ionic liquid composites. Adv. Mater. 22, 468–472 (2010). 20217735 10.1002/adma.200902213

[b34] Cheng Z., Wang T., Li X., Zhang Y. & Yu H. NIR-Vis-UV light-responsive actuator films of polymer-dispersed liquid crystal/graphene oxide nanocomposites. ACS Appl. Mater. Inter. 7, 27494–27501 (2015). 10.1021/acsami.5b09676 26592303

[b35] Yang H. . Thermally bandwidth-controllable reflective polarizers from (polymer network/liquid crystal/chiral dopant) composites. Appl. Phys. Lett. 82, 2407 (2003).

