# Supplementary material for: Unconventional High-Performance Laser Protection System Based on Dichroic Dye-Doped Cholesteric Liquid Crystals
Source: Sci Rep. 2017 Feb 23;7:42955. doi: 10.1038/srep42955 (PMC5322380; doi:10.1038/srep42955)
Supplement: Supplementary Information [file srep42955-s1.pdf]

Supplementary Information

Unconventional High-Performance Laser Protection System Based on Dichroic Dye-Doped Cholesteric Liquid Crystals

Wanshu Zhang[1], Lanying Zhang[2,3], Xiao Liang[2,3], Le Zhou[2,3], Jiumei Xiao[1], Li Yu[2,3], Fasheng Li[4], Hui Cao[1], Kexuan Li[5]*, Zhou Yang[1] and Huai Yang[1,2,3]*

[1]Department of Materials Physics and Chemistry, School of Materials Science and Engineering, University of Science and Technology Beijing. [2]Department of Materials Science and Engineering, College of Engineering, Peking University. [3]Key Laboratory of Polymer Chemistry and Physics of Ministry of Education, Peking University. [4]Department of Chemistry, Dalian Medical University. [5]Department of Applied Statistics and Science, Xijing University, Xian 710123, P. R. China

Supplementary Synthesis Procedures

All reactions were carried out under Argon. Dichloromethane and acetonitrile were distilled from CaH_2. Toluene was distilled from sodium and benzophenone. All chemicals were purchased from commercial sources and used as received, unless stated otherwise.

Synthesis of the dichroic yellow dye-D_1

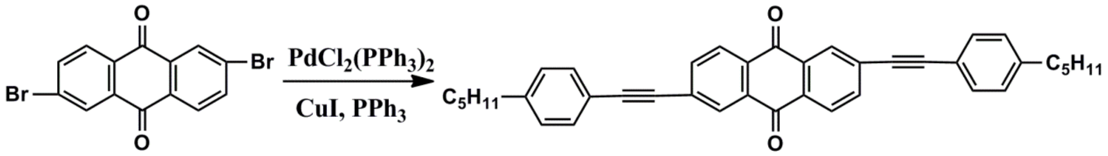

Supplymentray Figure S1. Synthesis of the dichroic yellow dye D_1.

2,6-Bis((4-pentylphenyl)ethynyl)anthracene-9,10-dione (D_1): The co-catalyst copper iodide (0.105 g, 0.546 mmol) was dissolved under Ar in a solution of

2,6-dibromoanthraquinone (1.00 g, 2.73 mmol) in anhyd toluene (20 mL). Then, anhyd Et_3N (20 mL), dichlorobis(tripenylphosphine)palladium (II) (0.115 g, 0.164 mmol), PPh_3 (0.043 g, 0.164 mmol) and 1-ethynyl-4-pentylbenzene (1.04 g, 6.01 mmol) were added under Ar and the mixture was refluxed for 12 h and poured into water (50 mL). The resulting mixture was extracted with dichloromethane (3 × 50 mL) and the combined organic layers were washed with water (3 × 50 mL), brine (2 × 50 mL), and dried over Na_2SO_4. After filtration the solvent was removed under reduced pressure to give 1.285 g of a yellow-brown solid. The crude product was purified by column chromatography over silica gel using (dichloromethane: petroleum mether = 1: 2) as the eluent. The product D_1 was isolated as a yellow solid (1.025 g, 68.3%). ^{1}H NMR (400 MHz, $CDCl_3$, δ): 8.43 (s, 2H, Ar-H), 8.30 (d, J = 8.0 Hz, 2H; Ar-H), 7.89 (d, J = 8.0 Hz, 2H; Ar-H), 7.50 (d, J = 8.0 Hz, 4H; Ar-H), 7.21 (d, J = 8.4 Hz, 4H; Ar-H), 2.64 (t, 4H, Ar-CH_2), 1.64 (m, 4H, CH_2), 1.35 (m, 8H, CH_2), 0.90 (t, 6H, CH_3); ^{13}C NMR (100 MHz, $CDCl_3$, δ): 182.20, 144.68, 136.42, 133.81, 131.93, 130.40, 128.93, 127.40, 119.11, 95.17, 87.59, 36.22, 31.44, 30.85, 22.67, 14.07. MS (MALDI-TOF, m/z): $[M+H]^+$ calcd for $C_{40}H_{36}O_2$, 548.2715; found, 550.3146. Anal. calcd for $C_{40}H_{36}O_2$: C 87.56, H 6.61, N 5.83; found: C 87.57, H 6.61, N 5.84.

Synthesis of the dichroic red dye-D_2

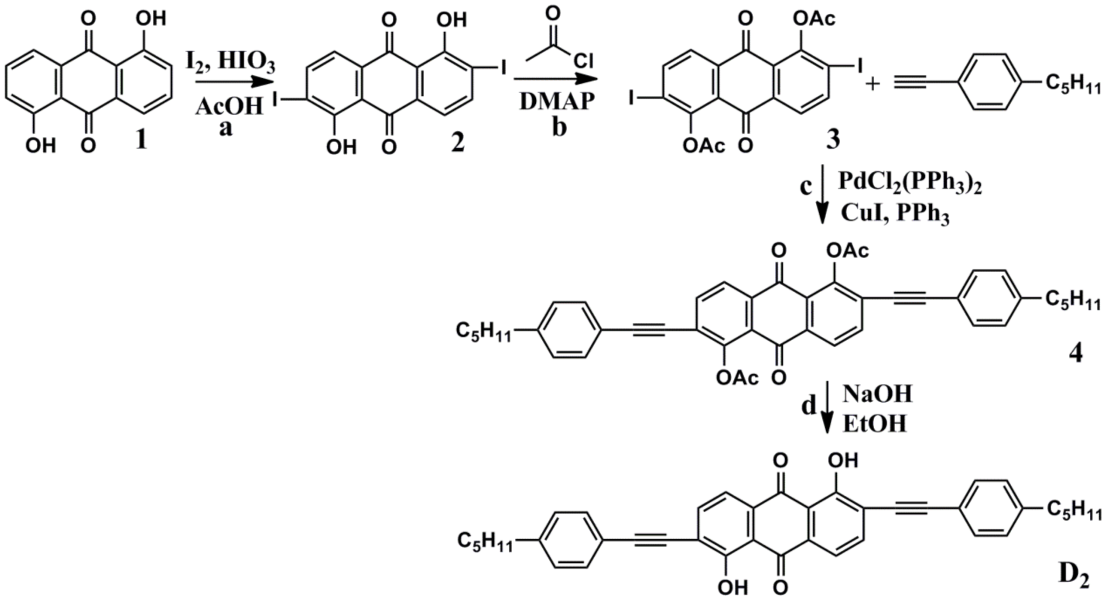

Supplymentray Figure S2. Synthesis of the dichroic red dye D_2.

1,5-Dihydroxy-2,6-diiodoanthracene-9,10-dione (2): H_2SO_4 (30%, 10 mL), I_2 (3 g, 11.8 mmol) and HIO_3 (1.375 g, 7.75 mmol) were added to a solution of 1,5-dihydroxyanthraquinone (1; 3.096 g, 12.90 mmol) in AcOH (150 mL). The mixture was refluxed for 6 h and, after cooling to room temperature, a yellow solid separated out on dilution with water (50 mL). The solid product was filtered, washed with saturated sodium thiosulfate solution to remove excess iodine and then with cold water. The dark-yellow crude product was dried in an oven and used for the next procedure without further purification.

2,6-Diiodo-9,10-dioxo-9,10-dihydroanthracene-1,5-diyl diacetate (3): To a solution of 2 (2.00 g, 4.07 mmol) and 4-dimethylaminopyridine (1.24 g, 10.16 mmol) in dry CH_2Cl_2 (40 mL) was added dropwise a solution of acetyl chloride (0.80 g, 10.16 mmol) in dry CH_2Cl_2 (10 mL) at 0 ^{o}C under Ar. The reaction mixture was refluxed for 4 h. Water (50 mL) was added and the mixture then extracted with dichloromethane. The combined organic extracts were washed with water and dried with $MgSO_4$, and

concentrated. The resulting precipitate was collected by filtration, washed with water and dried in an oven to give 1.6 g of a yellow solid.

9,10-Dioxo-2,6-bis((4-pentylphenyl)ethynyl)-9,10-dihydroanthracene-1,5-diyl diacetate (4): The co-catalyst copper iodide (0.105 g, 0.546 mmol) was dissolved under Ar in a solution of 3 (1.57 g, 2.73 mmol) in anhyd toluene (20 mL). Then, anhyd Et_3N (20 mL), dichlorobis(tripenylphosphine)palladium (II) (0.115 g, 0.164 mmol), PPh_3 (0.043 g, 0.164 mmol) and 1-ethynyl-4-pentylbenzene (1.04 g, 6.01 mmol) were added under Ar and the mixture was stirred at room temperature for 24 h and poured into water (50 mL). The resulting mixture was extracted with dichloromethane (3 × 50 mL) and the combined organic layers were washed with water (3 × 50 mL), brine (2 × 50 mL), and dried over Na_2SO_4. After filtration the solvent was removed under reduced pressure to give 1.285 g of a yellow-brown solid. The crude product was purified by column chromatography over silica gel using dichloromethane as the eluent. The product 4 was isolated as a yellow solid (0.98 g, 54.0%). ^{1}H NMR (400 MHz, $CDCl_3$, δ): 8.17 (d, J = 8.4 Hz, 2H; Ar-H), 7.92 (d, J = 8.0 Hz, 2H; Ar-H), 7.45 (d, J = 8.4 Hz, 4H; Ar-H), 7.20 (d, J = 8.0 Hz, 4H; Ar-H), 2.64 (t, 4H, Ar-CH_2), 2.55 (s, 6H, -OAc), 1.63 (m, 4H, CH_2), 1.34 (m, 8H, CH_2), 0.90 (t, 6H, CH_3).

1,5-Dihydroxy-2,6-bis((4-pentylphenyl)ethynyl)anthracene-9,10-dione (D_2): A solution of 4 (0.60 g, 0.90 mmol) in ethanol (30 mL) was refluxed for 5 h under nitrogen with 1 N NaOH (30 mL). The mixture was acidified by addition of dilute HCl (18 mL) and extracted with CH_2Cl_2 (30 mL). The organic phase was washed with

brine, dried (Na_2SO_4), filtered, and concentrated at reduced pressure. The crude product was purified by flash chromatography on a short column of the slightly acidified silica gel (dichloromethane: petroleum mether = 30: 1) and isolated as a red solid (0.22 g, 42.1%). ^{1}H NMR (400 MHz, $CDCl_3$, δ): 12.30 (s, 2H, -OH), 7.86 (s, 4H, Ar-H), 7.53 (d, J = 8.4 Hz, 4H; Ar-H), 7.20 (d, J = 8.0 Hz, 4H; Ar-H), 2.64 (t, 4H, Ar-CH_2), 1.59 (m, 4H, CH_2), 1.30 (m, 8H, CH_2), 0.90 (t, 6H, CH_3); ^{13}C NMR (100 MHz, $CDCl_3$, δ): 186.59, 162.27, 143.86, 138.23, 131.17, 127.58, 119.89, 118.66, 117.83, 115.14, 99.09, 82.48, 35.10, 30.68, 29.78, 21.74, 12.84; MS (MALDI-TOF, m/z): $[M+H]^+$ calcd for $C_{40}H_{36}O_4$, 580.2614; found, 582.2890. Anal. calcd for $C_{40}H_{36}O_4$: C 82.73, H 6.25, O 11.02; found: C 87.75, H 6.26, N 11.02.

Synthesis of the contrastive blue dye-D_C:

The contrastive blue dye D_C was synthesized according to the reference 1. Synthesis procedure is as follows:

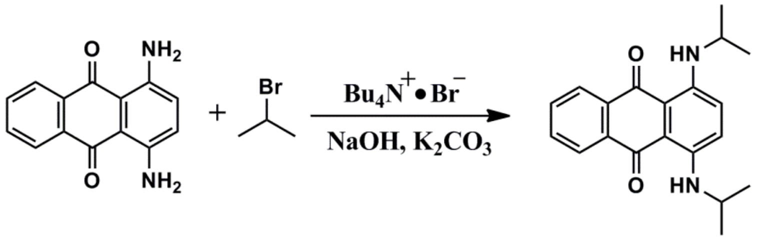

Supplymentray Figure S3. Synthesis of the contrastive blue dye D_C.

1,4-Bis(isopropylamino)anthracene-9,10-dione (D_C): A mixture of 1,4-diamino anthraquinone (2.358 g, 0.01 mol), powdered sodium hydroxide (3.20 g, 0.08 mol), anhydrous potassium carbonate (2.76 g, 0.02 mol) and tetrabutylammonium bromide (0.124 g, 0.4 mmol) in dichloroethane (10 mL) was stirred at 30 ^{o}C for 1 h. Then the alkylating agent 2-bromopropane (2.583 g, 0.021 mol) was added dropwise over a

period of 30 min. The reaction mixture was heated at 50 ^{o}C for 10 h. At the end the inorganic material was filtered and washed with 10 mL dichloroethane. The organic phase was washed with water (2 × 10 mL), dried (Na_2SO_4), and concentrated at reduced pressure. The crude product was purified by column chromatography over silica gel using dichloromethane as the eluent. The product D_C was isolated as a blue solid (1.24 g, 38.5%). ^{1}H NMR (400 MHz, $CDCl_3$, δ): 10.84 (s, 2H, Ar-NH), 8.33 (q, J = 8.8 Hz, 2H; Ar-H), 7.71 (q, J = 9.2 Hz, 2H; Ar-H), 7.33 (s, 2H; Ar-H), 3.93 (s, 2H, -CH-), 1.40 (d, 12H, $-CH_3$); ^{13}C NMR (100 MHz, $CDCl_3$, δ): 182.49, 145.28, 134.53, 132.00, 12.98, 124.10, 110.30, 44.77, 23.34. MS (MALDI-TOF, m/z): $[M+H]^+$ calcd for $C_{20}H_{22}N_2O_2$, 322.1681; found, 322.1387. Anal. Calcd for $C_{20}H_{22}N_2O_2$: C 74.51, H 6.88, N 8.69, O 9.93; found: C 74.54, H 6.89, N 8.69, O 9.94.

a

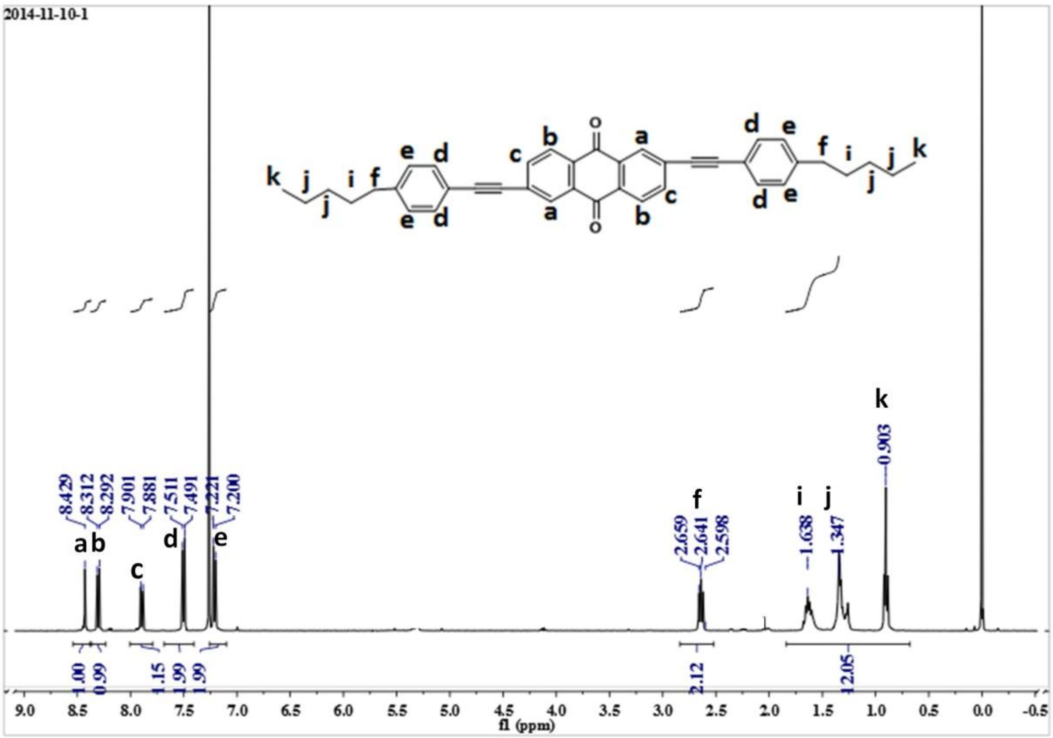

b

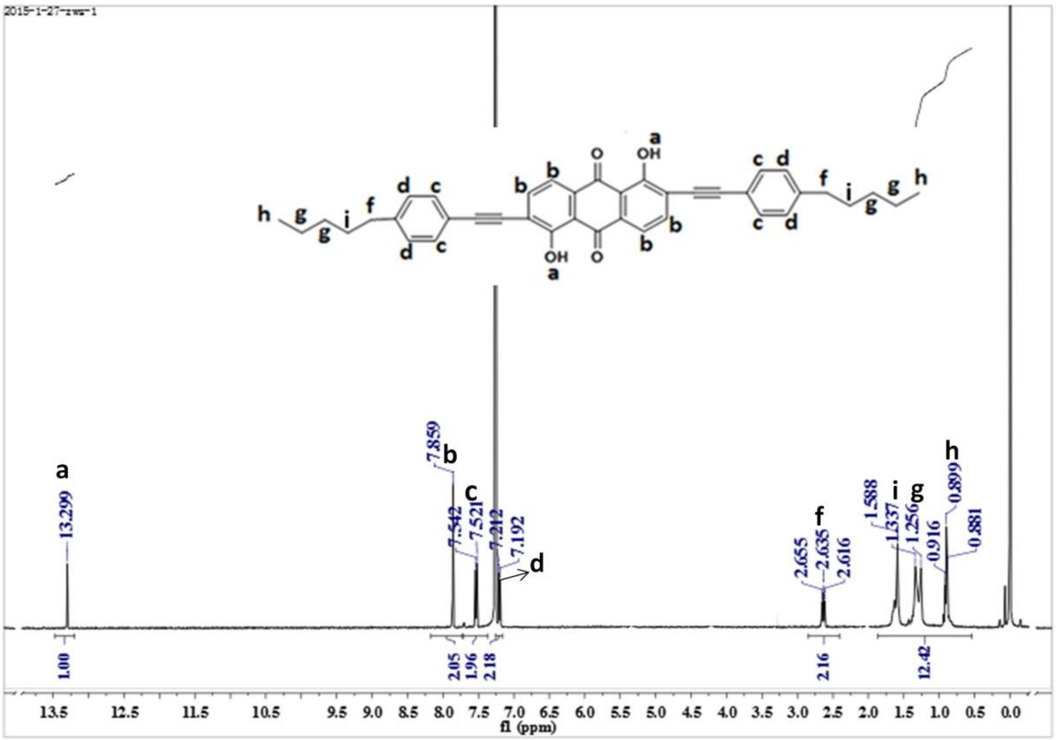
2015-1-27-zws-1
13.299
7.859
7.542
7.521
7.212
7.192
2.655
2.635
2.616
1.588
1.337
1.256
0.916
0.899
0.881
1.00
2.05
1.96
2.18
2.16
12.42
f1 (ppm)

c

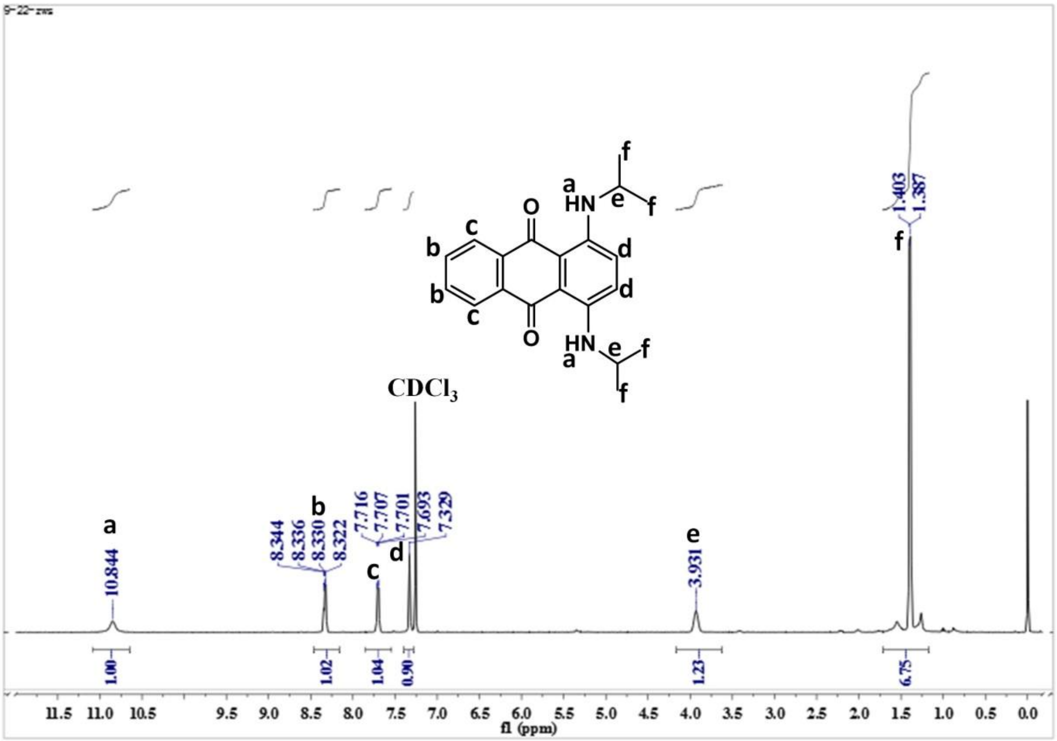
9-22-zws
10.844
8.344
8.336
8.330
8.322
7.716
7.707
7.701
7.693
7.329
3.931
1.403
1.387
CDCl$_3$
1.00
1.02
1.04
0.90
1.23
6.75
f1 (ppm)

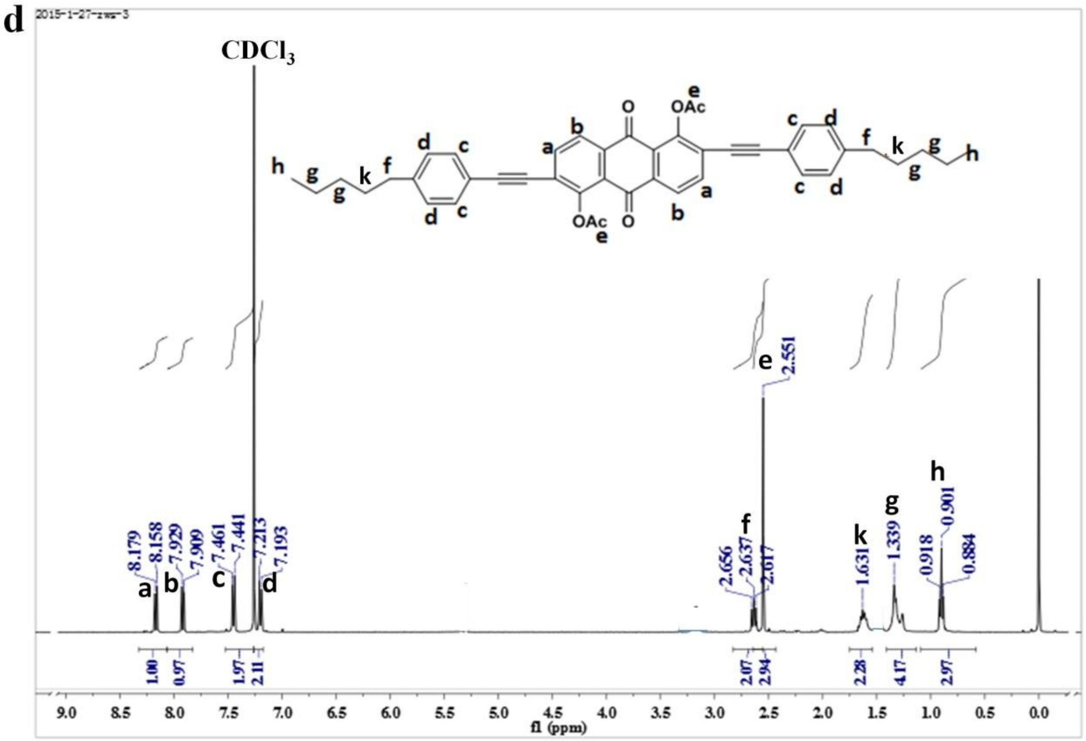

Supplymentray Figure S4. ^{1}H NMR spectra of the AQ dyes (a) D_1, (b) D_2, (c) D_C and (d) intermediate dye 4.

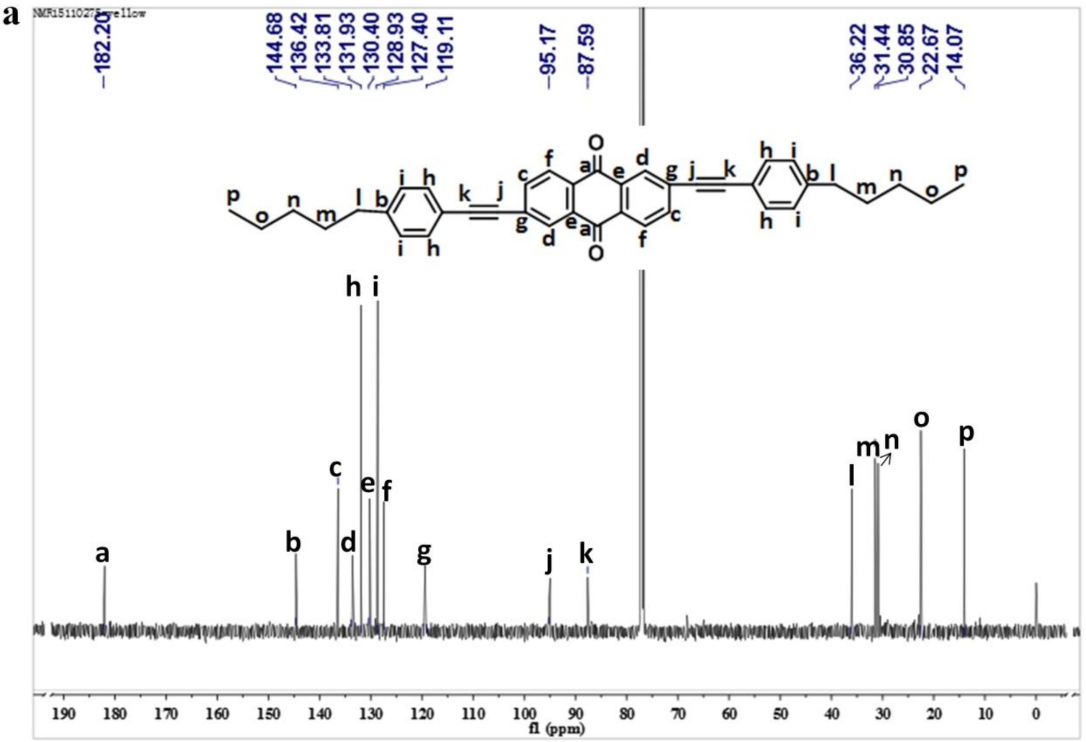

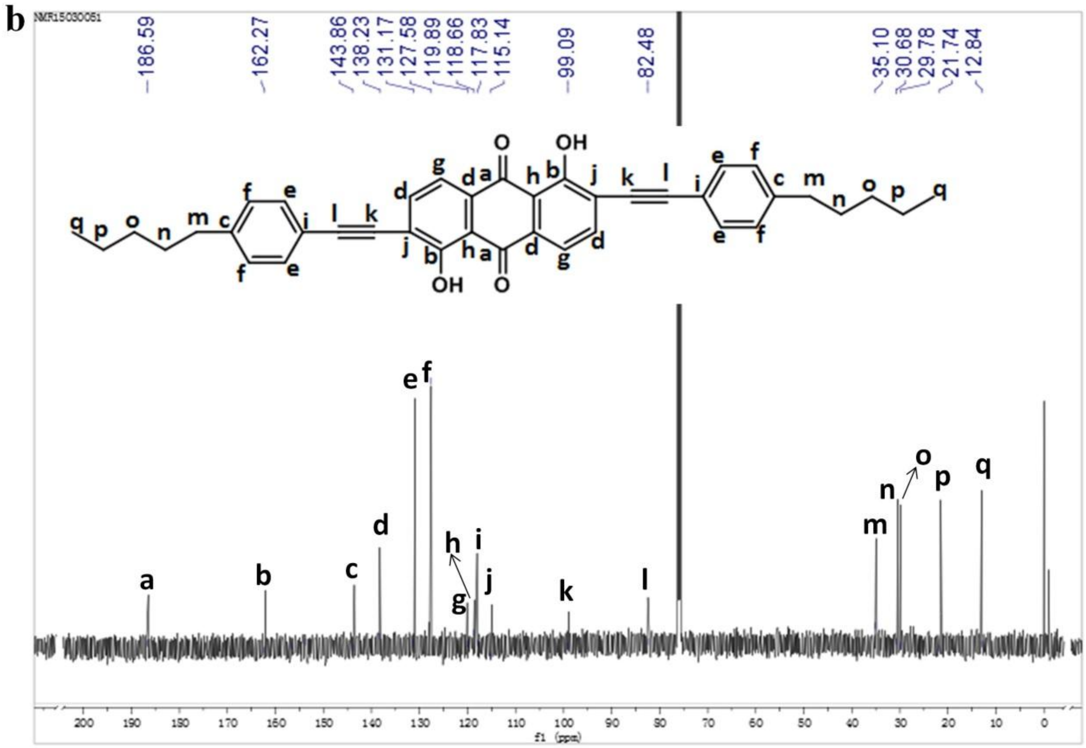

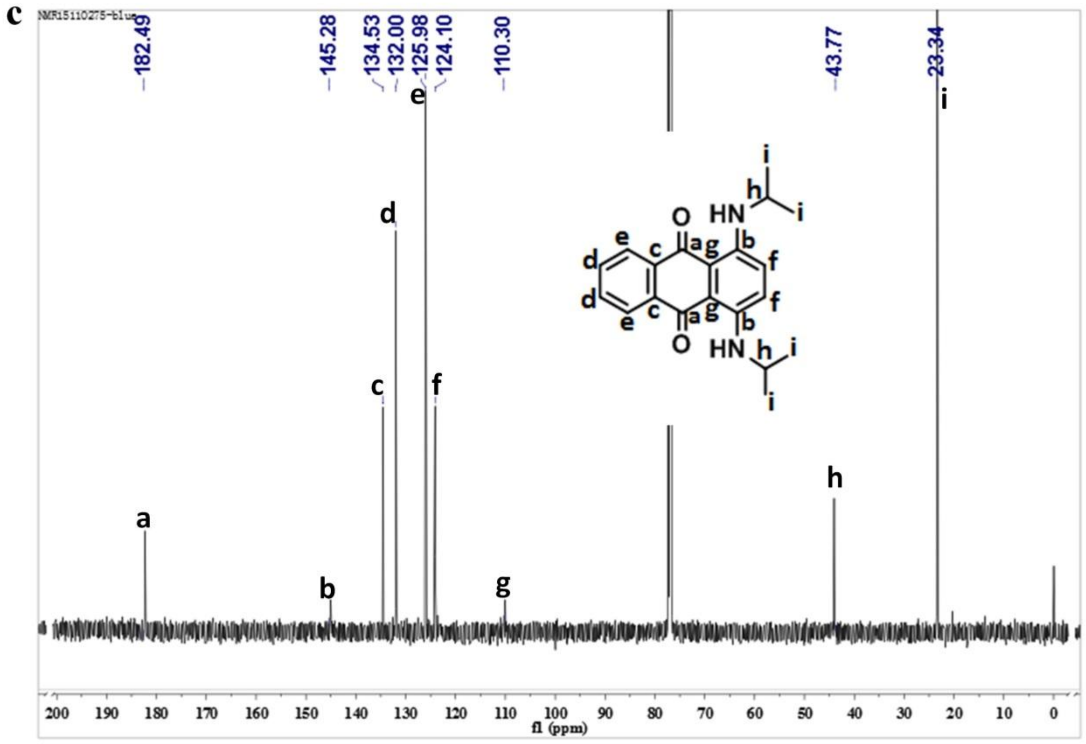

Supplymentray Figure S5. ^{13}C NMR spectra of the AQ dyes (a) D_1, (b) D_2, (c) D_C.

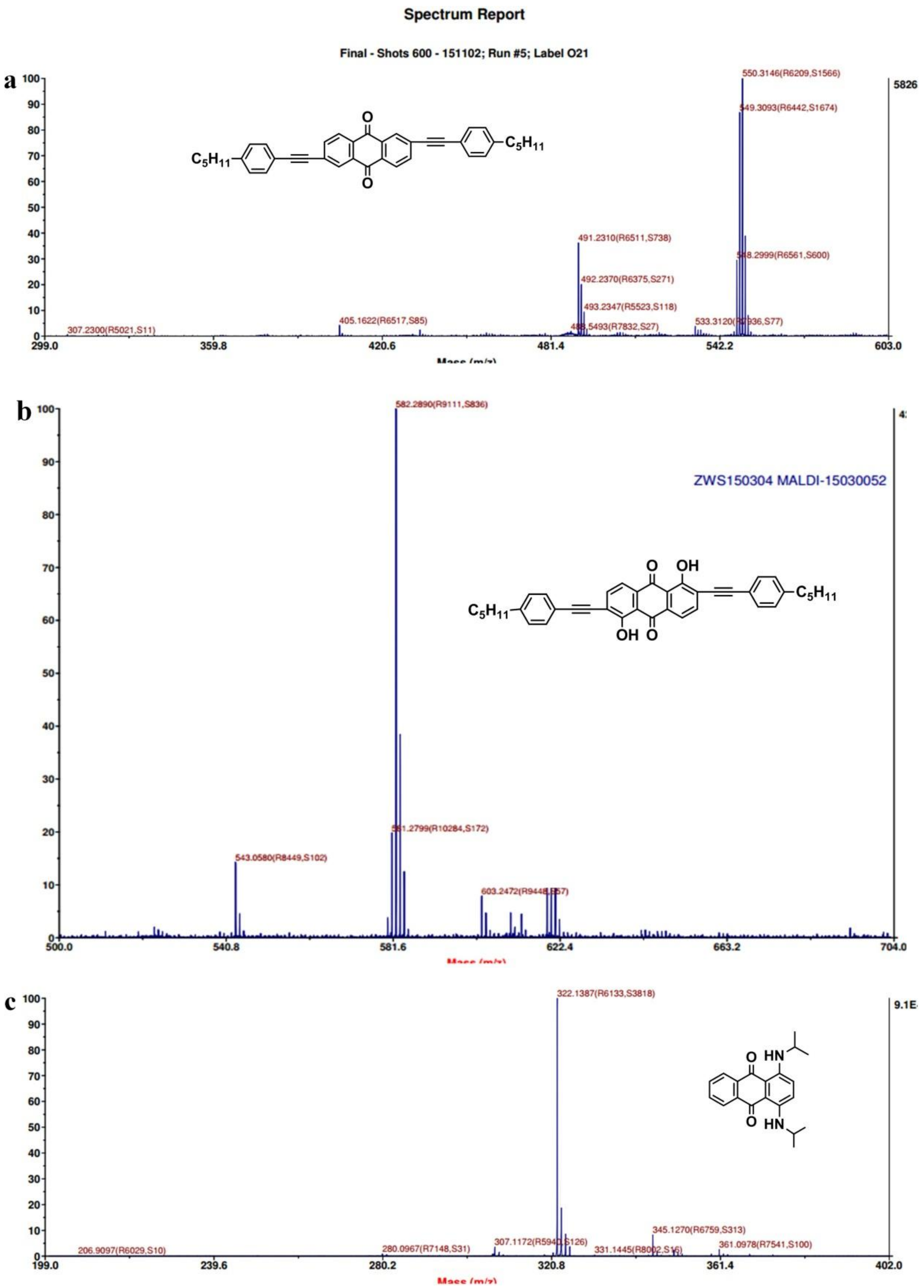

Supplymentray Figure S6. MALDI-TOF MS spectra of the AQ dyes (a) D_1, (b) D_2 and (c) D_C.

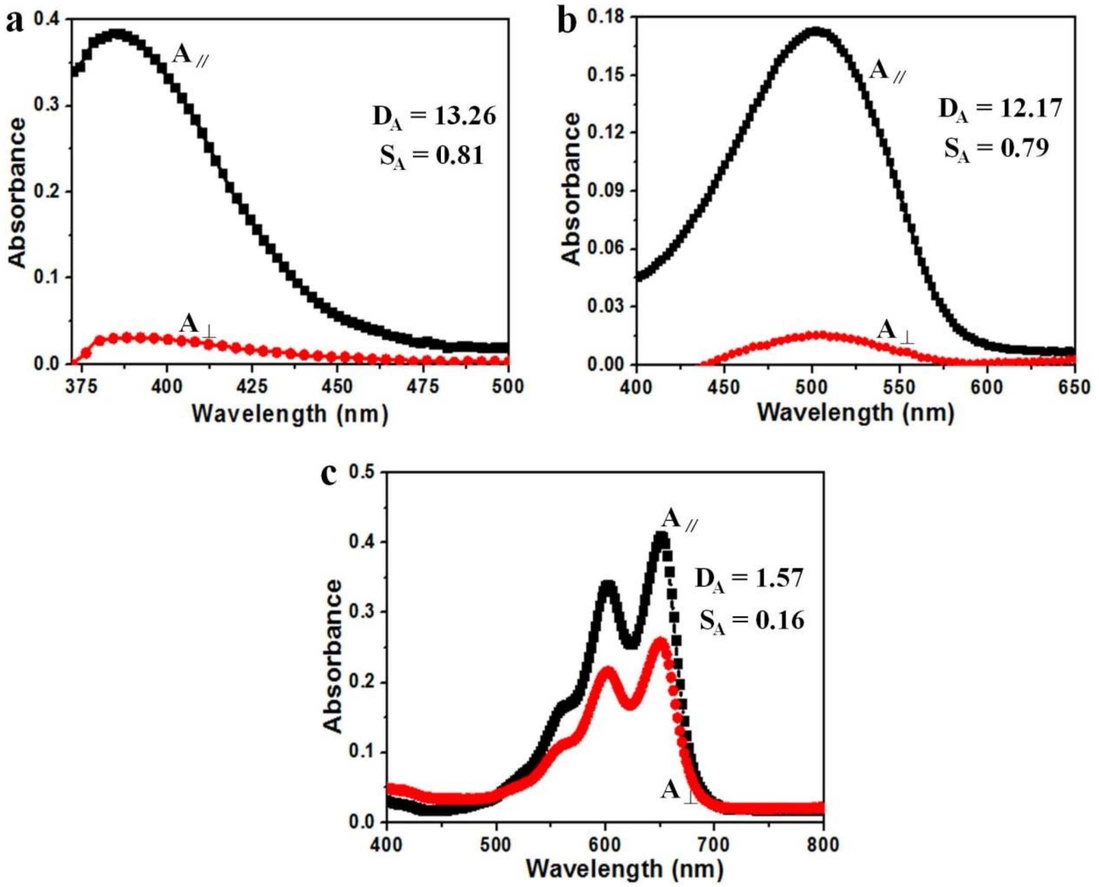

Supplymentray Figure S7. Polarized absorption spectra of AQ dyes. (**a**) D_1 (**b**) D_2 and (**c**) D_C dissolved in LC E7 at a concentration of 0.5% w/w for D_1, D_2 and 0.8% w/w for D_C.

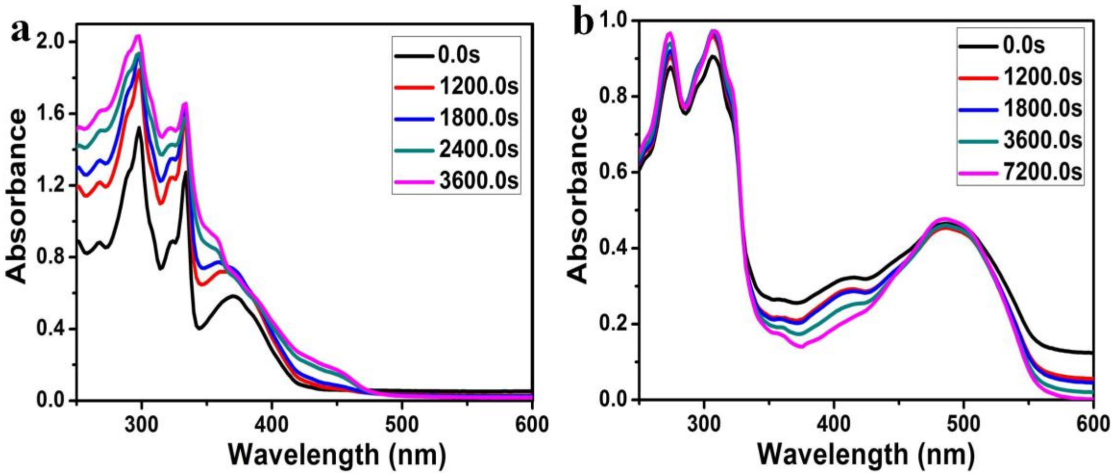

Supplymentray Figure S8. **UV-stability measurement of the dichroic dyes**. UV-vis absorption spectra of (**a**) D_1 and (**b**) D_2 dissolved in the UV-transparent

cyclohexane solution upon 365 nm UV irradiation (50.0 mW/cm^2). Although the working wavelengths for dichroic dyes D1 and D2 series are 420 nm and 500 nm, the samples are prepared via photopolymerization under 365 nm UV light of 5.0 mW/cm^2 intensity for 600.0 s. It is of great importance to disscuss about the dye UV stability, which depends on two main factors of absorption and molecular structure. D1 and D2 have the same molecular skeleton structure of bistolane, hence, we focus on the absorption factor and measure the UV-vis absorption spectra of D_1 and D_2 in the UV-transparent cyclohexane solution upon 365 nm UV irradiation (50.0 mW/cm^2).

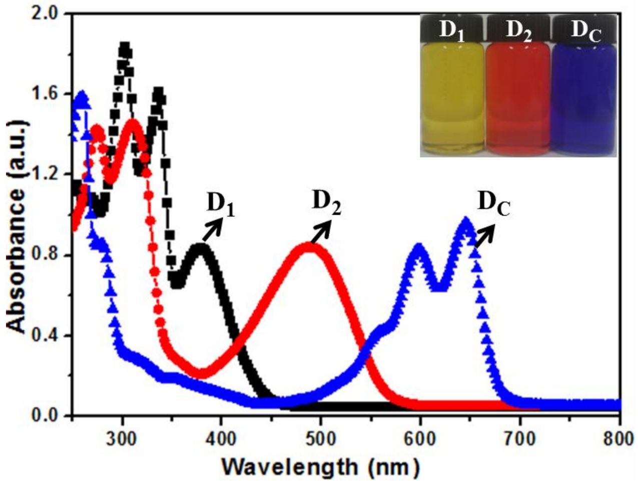

Supplymentray Figure S9. **UV-vis absorption spectra of D_1, D_2 and D_C in methylbenzene at 25 ^{o}C.** The UV-vis absorption spectra of AQ dyes exhibit progressive bathochromic shifts (compared with D_1, D_2 and D_C redshift 113 and 222 nm, respectively), the extent of which can be attributed to the inductive effect of the electron-donating substitutes(-NH->-OH>-H) on the AQ cores.[2] The absorption peak around 300 nm is attributed to the n–π* transition of the AQ backbones, while the prominent charge transfer absorption in the visible region is due to the π-π*

transition.[3] Furthermore, the three AQ dyes assimilate the primate trichromatic colours and collectively shield the light over a wide range of 250-700 nm.

In this study, non-reactive nematic liquid crystals (LCs), HNG726200-100; chiral dopants, S1011 and R1011; a photo-polymerisable nematic monomer, C6M; and a photoinitiator, 2, 2-dimethoxy-2-phenyl-acetophenone, Irgacure 651, are used with the material details shown in Supplymentray Fig. 9. In laser protection experiments, for each dye series (D_1, D_2 and D_C), there are three samples of CLC reflection sample, the dye absorption sample and the corresponding dye-doped CLC complex sample with the specific formulas shown in Supplymentray Table 1, 2 and 3, respectively. The CLC sample is prepared by mixing HNG726200-100 with a left-handed chiral dopant S1011. By controlling the mixing ratio, the desired helical pitch, i.e., the reflection wavelength of CLCs is obtained to be in match with that of the dye absorption. The dye absortion sample is prepared by doping AQ dye in LC HNG726200-100 with C6M and Igacure 651 for stabilization. For the dye-doped CLC sample, dye, C6M, Igacure 651 and CLC mixtures are well stirred at 60 degree to prepare dye-doped CLC mixtures.

(1) Nematic LC:

HNG726200-100 (Jiangsu Hecheng Display Technology Co., Ltd.)

Mixture of LCs with negative dielectric anistropy (Δn = 0.255, ne =1.753, no =1.498, $\Delta\varepsilon$ = -4.0, $\varepsilon_{//}$ = 4.2, $\varepsilon_{\perp}$=8.2)

E7 (Jiangsu Hecheng Display Technology Co., Ltd.)

Mixture of LCs with positive dielectric anistropy (Δn = 0.199, ne =1.720, no =1.521, $\Delta\varepsilon$ = 11.5, $\varepsilon_{//}$ = 16.4, $\varepsilon_{\perp}$= 4.9)

(2) Chiral Dopants: S1011 and R1011 (Beijing Bayishikong LCD Technology Co., Ltd)

S1011 (Left-handed)

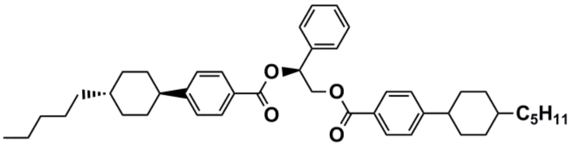

R1011 (Right-handed)

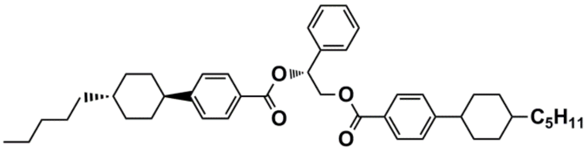

(3) Photo-polymerizable LC monomer: C6M

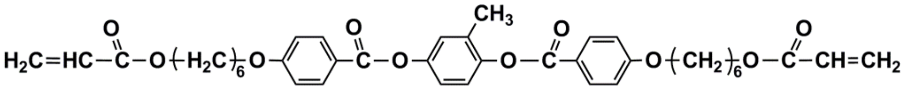

(4) Photeintiator (Igacure 651) 2,2-dimethoxy-1,2-diphenyl-ethanone (TCI Co., Ltd.)

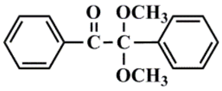

Supplymentray Figure S10. Chemical structures of the materials used.

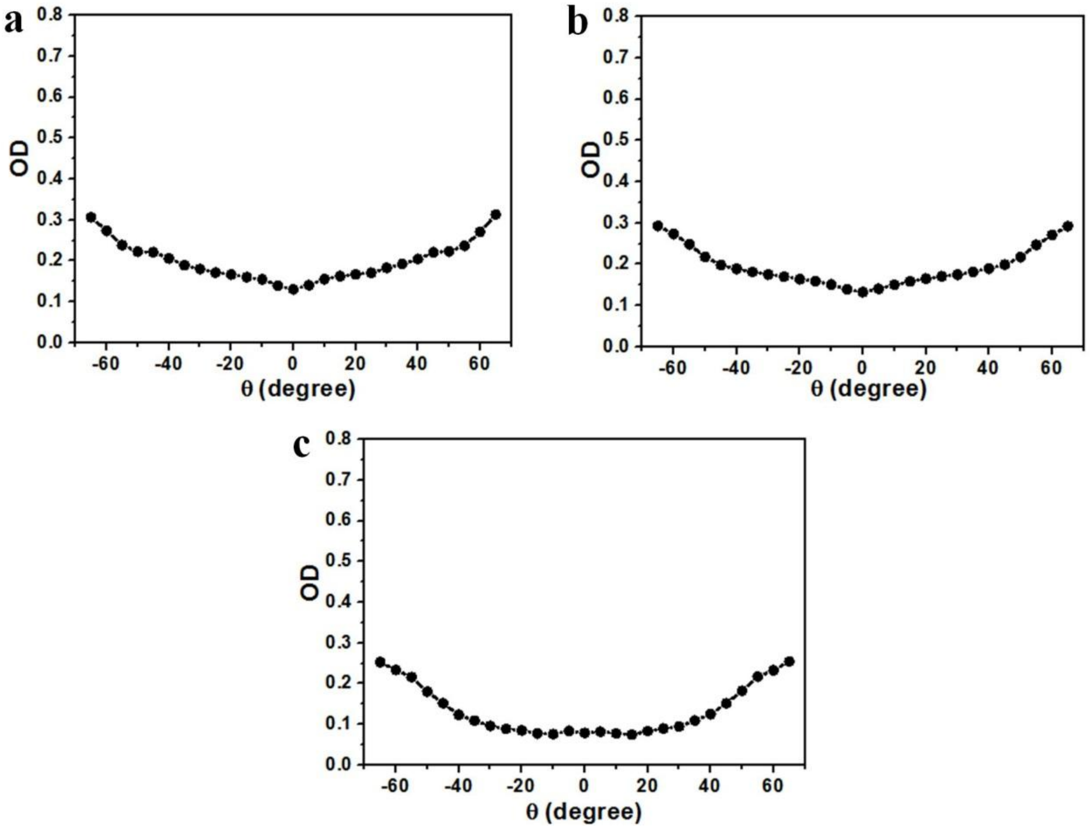

Supplymentray Figure S11. **OD measurements with different incident angles of empty LC cells at different wavelengths**. (**a**) 420 nm, (**b**) 500 nm, and (**c**) 600 nm.

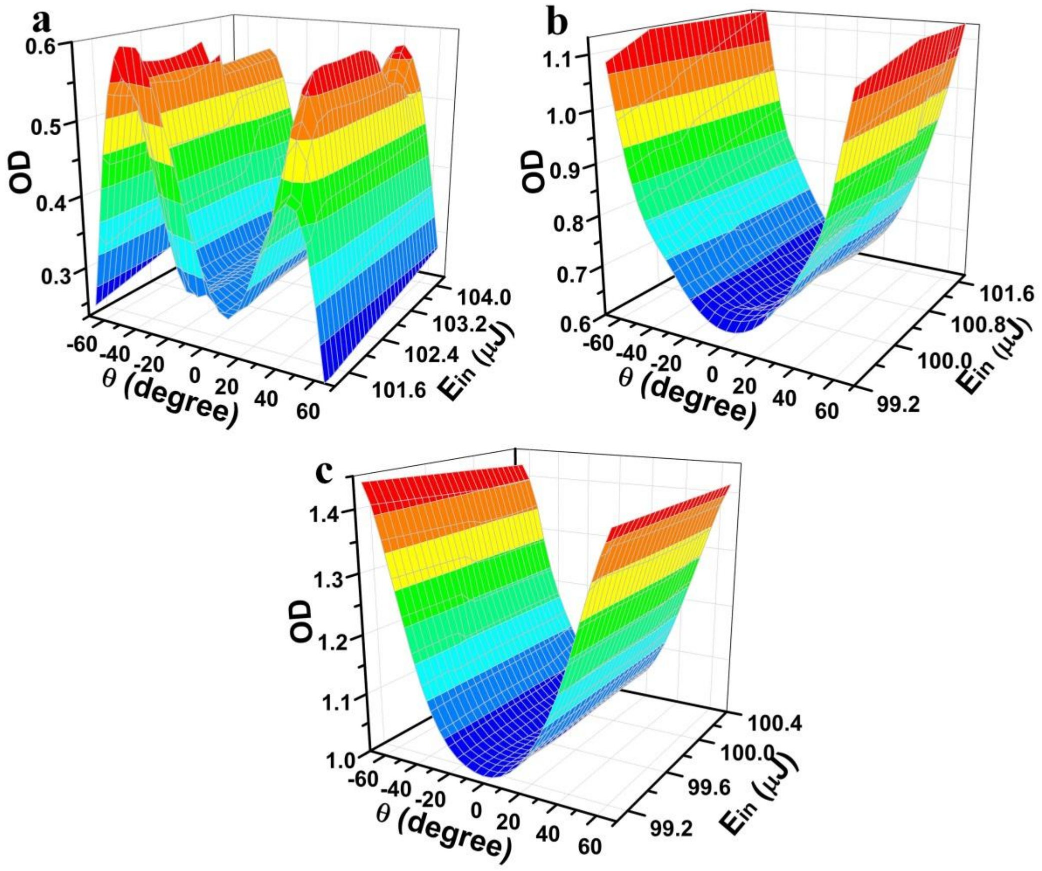

Supplymentray Figure S12. **Laser protection measuremen**t. Three-dimensional figures of OD measurements as a function of θ and E_{in} (Power density of 3.18×10^{-3} J/cm^2) of D_2 series. (**a**) CLC reflection, (**b**) D_2 absorption, and (**c**) D_2-doped CLC samples. The LC cells are all 20 μm thick. All the figures are the results removing the influence of empty LC cells (Supplymentray Fig. S11).

Supplymentray Table S1. Formulas of D_1 series for UV-Vis spectra and laser protection measurement

Num	HNG726200-100	S1011	C6M	D_1	651
1	85.8	14.2	0.0	0.0	0.0
2	99.0	0.0	0.0	1.0	0.0
3	76.5	13.5	8.0	1.0	1.0
4	90.0	0.0	8.0	1.0	1.0

Supplymentray Table S2. Formulas of D_2 series for UV-Vis spectra and laser protection measurement

Num	HNG726200-100	S1011	C6M	D_2	651
1	87.9	12.1	0.0	0.0	0.0
2	99.0	0.0	0.0	1.0	0.0
3	78.2	11.8	8.0	1.0	1.0
4	90.0	0.0	8.0	1.0	1.0

Supplymentray Table S3. Formulas of D_C series for UV-Vis spectra and laser protection measurement

Num	HNG726200-100	S1011	C6M	D_C	651
1	90.1	9.9	0.0	0.0	0.0
2	99.5	0.0	0.0	0.5	0.0
3	80.8	9.7	8.0	0.5	1.0
4	90.5	0.0	8.0	0.5	1.0

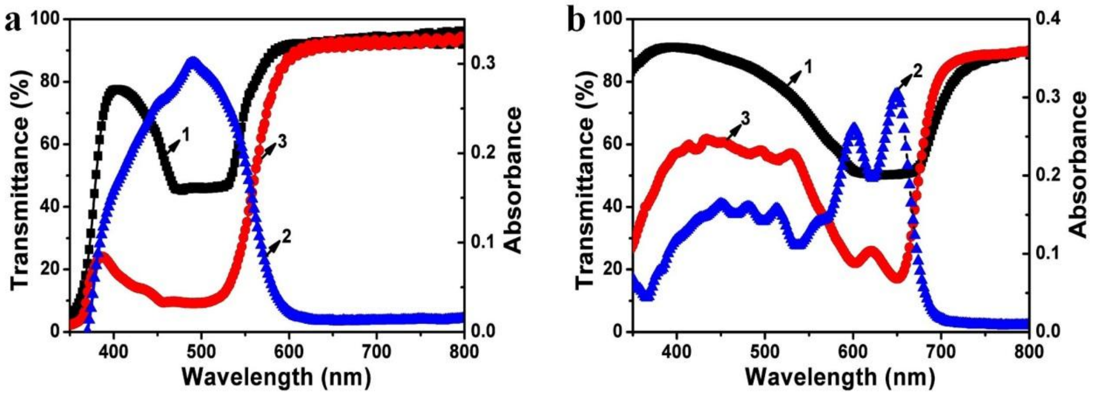

Supplymentray Figure S13. **UV-vis spectra of (a) D_2 and (b) D_C series.** 1 is CLC transmission spectrum, 2 is dye absorption spectrum and 3 is the corresponding dye-doped CLC transmission spectrum. The LC cells are all 20 μm thick. The sample Fomulas are given in Supplymentray Table 1-3. It should be noted that the dye

absorption samples for OD measurements are further stablized with C6M with the formulas given as “4” in each table.

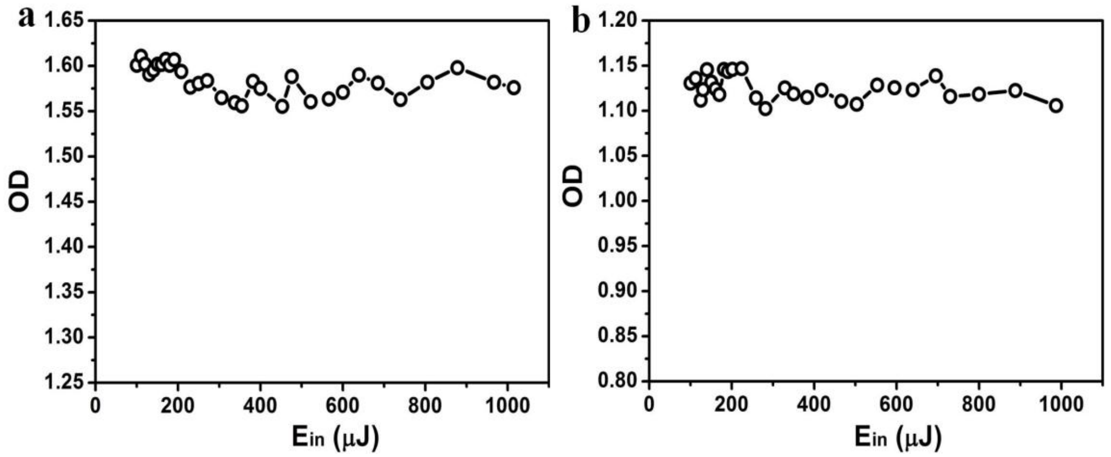

Supplymentray Figure S14. **OD measurement at the normal angle under different incident energies E_{in}.** (**a**) D_1-doped CLC complex sample and (**b**) D_2-doped CLC complex sample. The LC cells are 20 μm thick.

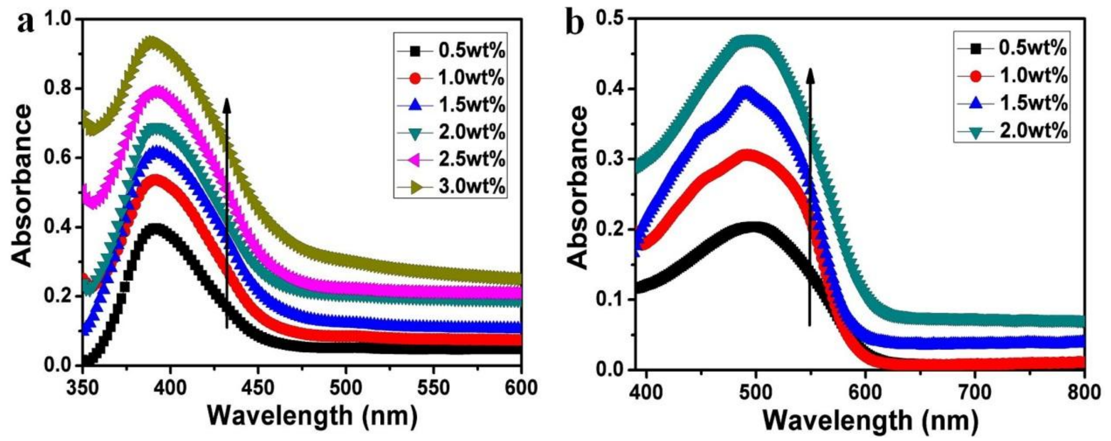

Supplymentray Figure S15. **Absorption spectra for dichroic dyes in different concentrations.** (**a**) D_1 with concentrations from 0.5-3.0 wt% and (**b**) D_2 with concentrations from 0.5-2.0 wt% in LC HNG726200-100 measured in 20 μm thick LC cells at 25 ^{o}C.

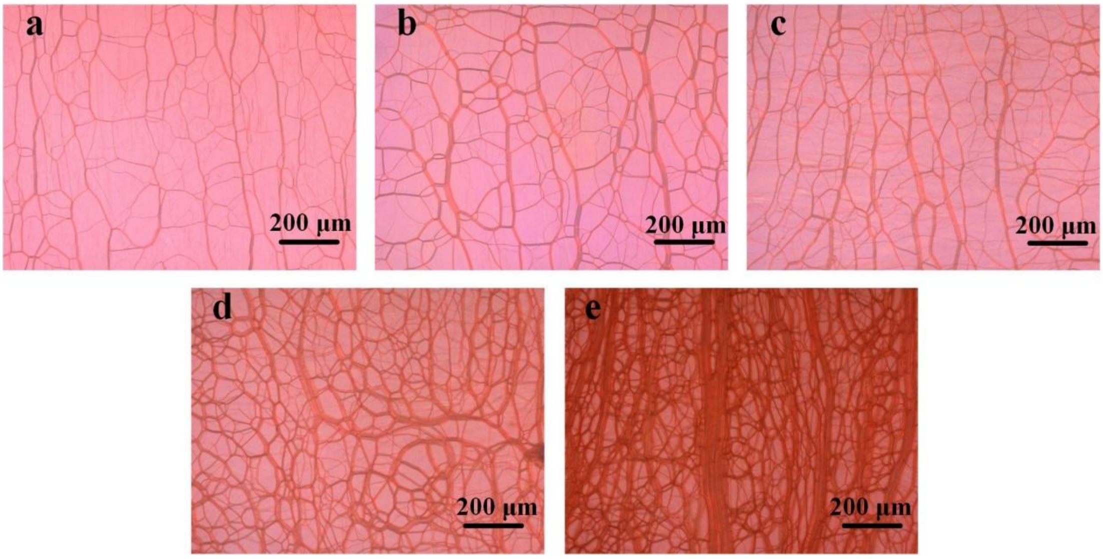

Supplymentray Figure S16. **POM photographs for CLC reflection samples in LC cells of different thicknesses**. (**a**) 20 µm, (**b**) 40 µm , (**c**) 60 µm, (**d**) 80 µm and (**e**) 100 µm.

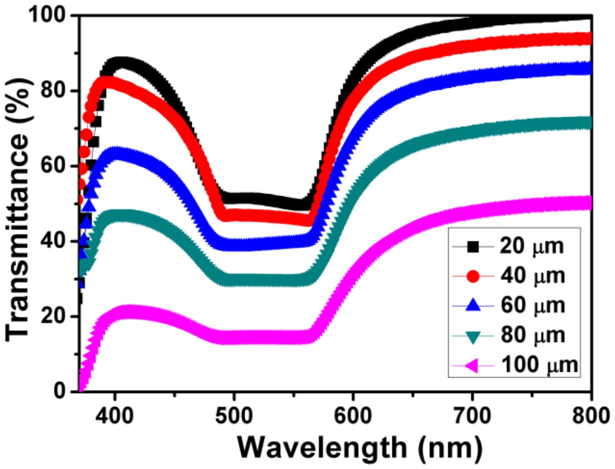

Supplymentray Figure S17. **UV-vis transmittance measurement**. Transmission spectra for CLC reflection samples in LC cells of different thicknesses of 20 µm, 40 µm , 60 µm, 80 µm and 100 µm.

Supplymentray Table S4. Formulas of the improved dye-doped single-handed CLC systems

Num	HNG726200-100	S1011	C6M	D_1	D_2	651
1	75.2	13.4	8.0	2.3	0.0	1.0
2	77.6	11.8	8.0	0.0	1.6	1.0

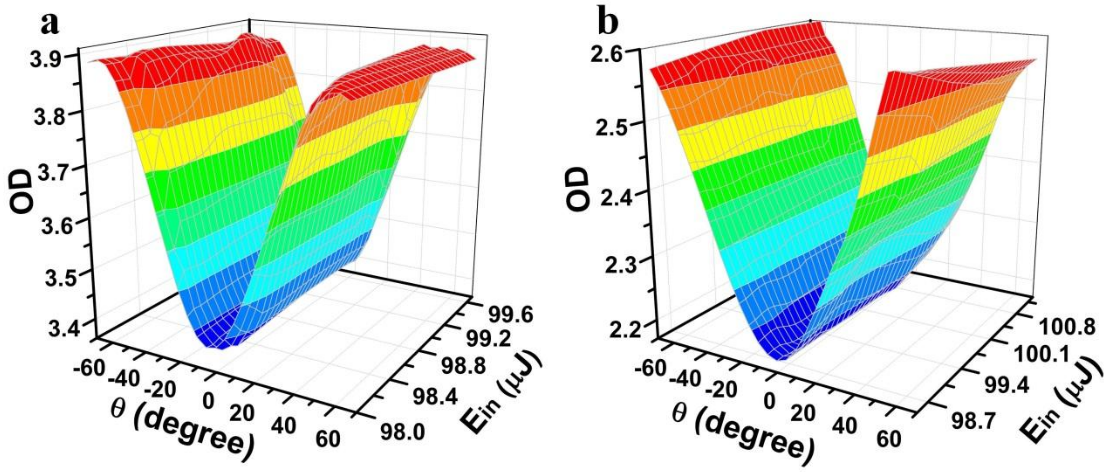

Supplymentray Figure S18. **Laser protection measuremen**t. Three-dimensional figures of OD measurement as a function of θ and E_{in} (Power density of 3.18×10^{-3} J/cm^2) for the improved dye-doped single-handed CLC samples of (**a**) D_1 series and (**b**) D_2 series in 60 μm thick LC cells. All the figures are the results removing the influence of empty LC cells (Supplymentray Fig. S11).

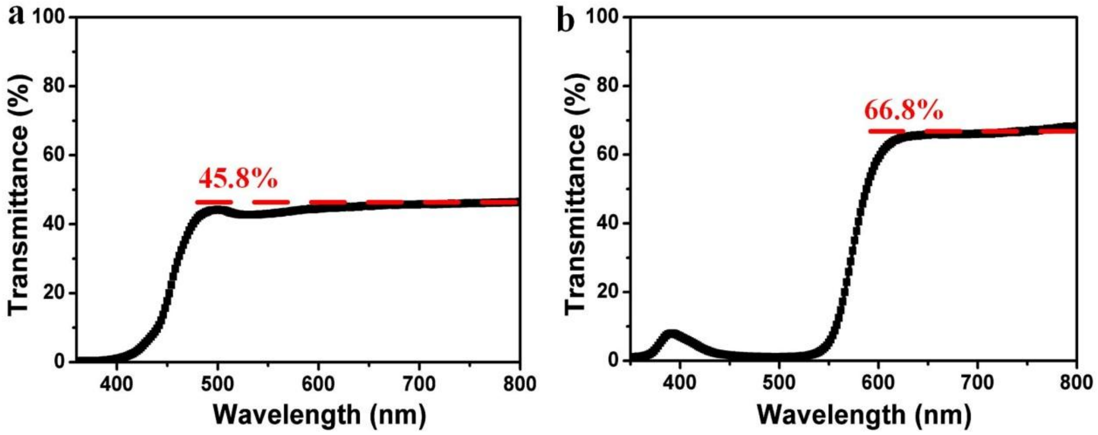

Supplymentray Figure S19. **Transmission spectra of the improved dye-doped single-handed CLC samples**. (**a**) D_1 series and (**b**) D_2 series in 60 μm thick LC cells.

Supplymentray Table S5. Formulas of the D_1-doped double-handed CLC systems

Num	HNG726200-100	S1011	R1011	C6M	D_1	651
1	75.4	13.7	0.0	8.0	0.0	1.0
2	84.0	0.0	13.7	0.0	2.3	0.0

Supplymentray Table S6. Formulas of the D_2-doped double-handed CLC systems

Num	HNG726200-100	S1011	R1011	C6M	D_2	651
1	79.1	11.9	0.0	8.0	0.0	1.0
2	86.4	0.0	12.0	0.0	1.6	0.0

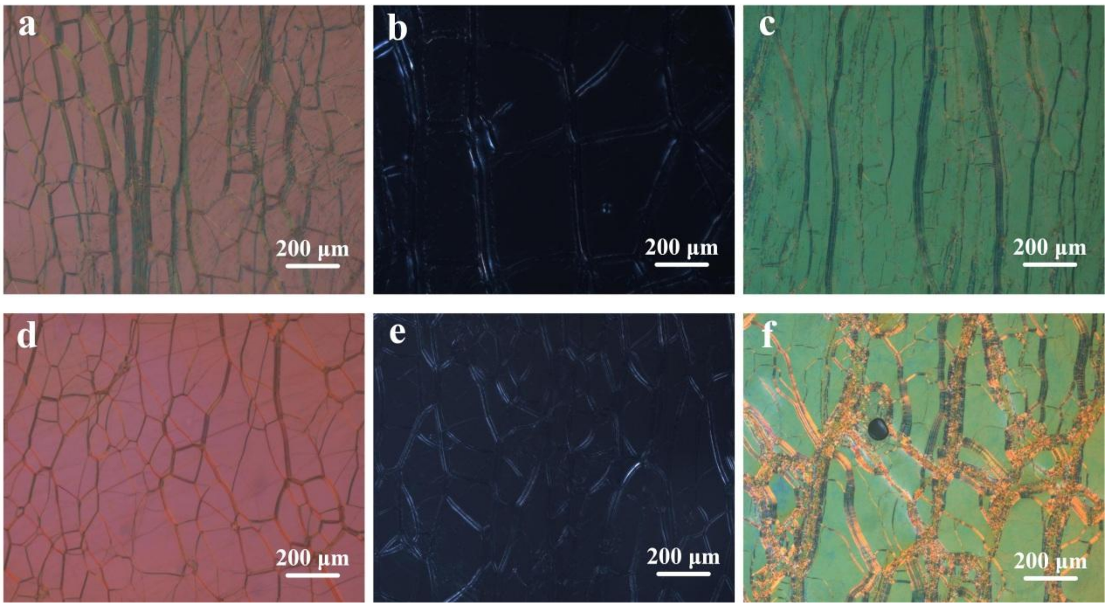

Supplymentray Figure S20. **POM photographs of samples in washout/refill method**. (**a-c**) D_1 series and (**d-f**) D_2 series. (**a**) and (**d**) are samples with left-handed helical structures; (**b**) and (**e**) are the samples with the nonreactive LCs washed out; (**c**) and (**f**) are the samples refilled with right-handed CLC and dichroic dye mixtures.

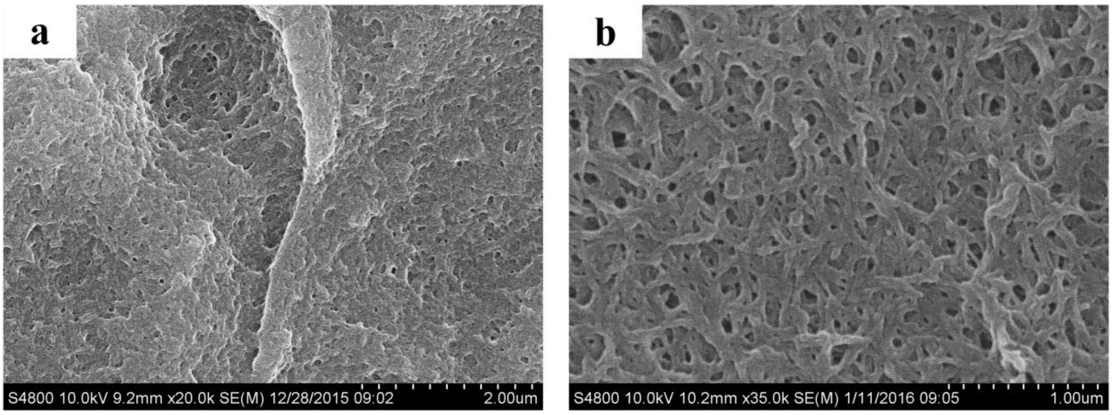

Supplymentray Figure S21. **SEM images of the prefabricated polymer network surface morphology in washout/refill method**. (**a**) D_1 series and (**b**) D_2 series (Formula 1 in Supplymentray Table 5 and 6, respectively).

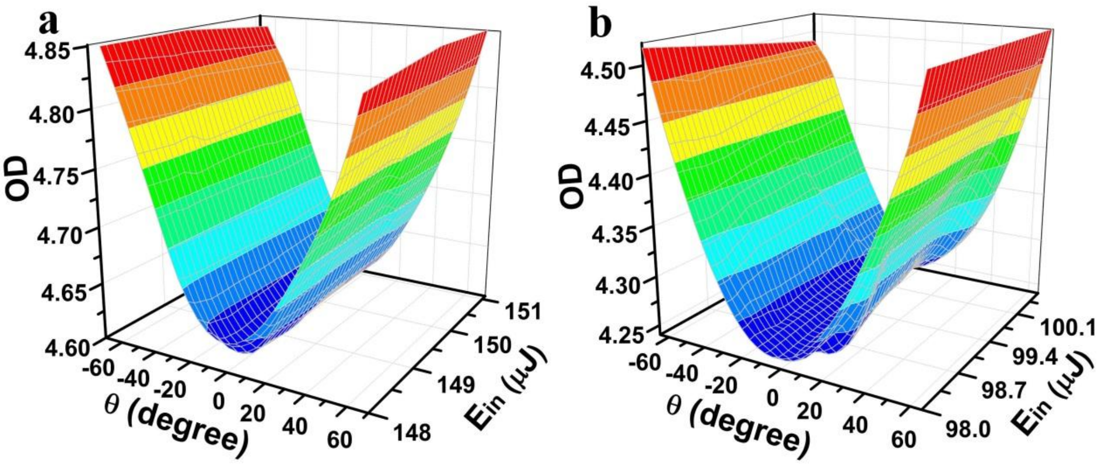

Supplymentray Figure S22. **Laser protection measuremen**t. Three-dimensional figures of OD measurements as a function of θ and E_{in} for the dye-doped double-handed CLC samples of (**a**) D_1 and (**b**) D_2 series. The power densities for D1 and D2 series are 4.78×10^{-3} J/cm^2 and 3.18×10^{-3} J/cm^2, respectively.

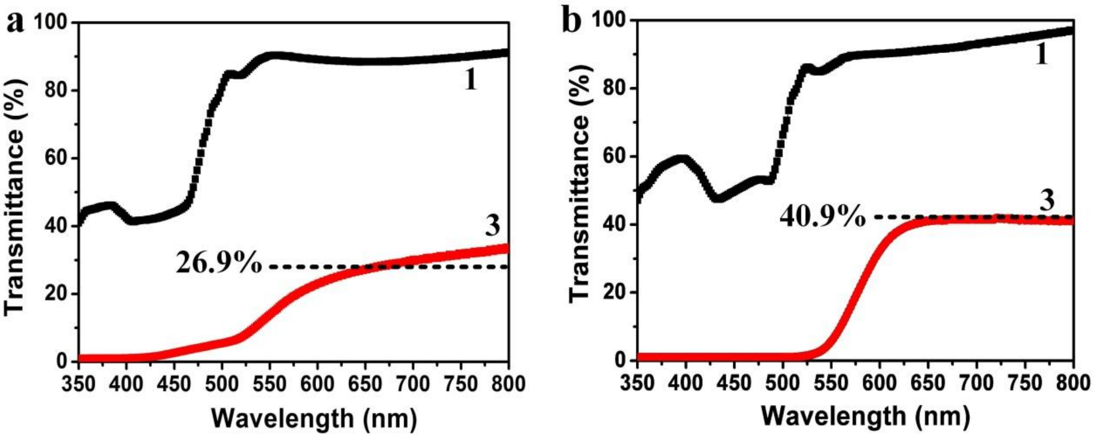

Supplymentray Figure S23. **Transmission spectra of samples in washout/refill method.** (**a**) D_1 and (**b**) D_2 series. 1 is the prefabricating left-handed sample and 3 is the corresponding dye-doped double-handed CLC sample. The LC cells are all 60 μm thick.

References

1. Ramrao, K. U., Ramkumar, C. A., Anant, N. A., Ramanuja, A. N. Phase-transfer catalyzed N-monoalkylation of aminoanthraquinone. *Syn. Commun*. **21**, 1129-1135 (1991).

2. Son, H. J. *et al*. Synthesis of fluorinated polythienothiophene-co-benzodithiophenes and effect of fluorination on the photovoltaic properties. *J. Am. Chem. Soc.* **133**, 1885-1894 (2011).

3. Itoh, T. Low-lying electronic states, spectroscopy, and photophysics of linear para acenequinones. *Chem. Rev*. **95**, 2351-2368 (1995).
